# Preparation and Neutralization Efficacy of Novel Jellyfish Antivenoms against *Cyanea nozakii* Toxins

**DOI:** 10.3390/toxins13020165

**Published:** 2021-02-21

**Authors:** Rongfeng Li, Huahua Yu, Aoyu Li, Chunlin Yu, Pengcheng Li

**Affiliations:** 1 Key Laboratory of Experimental Marine Biology, Center for Ocean Mega-Science, Institute of Oceanology, Chinese Academy of Sciences, Qingdao 266071, China; yuhuahua@qdio.ac.cn (H.Y.); aoyulee1227@163.com (A.L.); Yuchunlin17@mails.ucas.ac.cn (C.Y.); 2 Laboratory for Marine Drugs and Bioproducts, Pilot National Laboratory for Marine Science and Technology (Qingdao), No. 1 Wenhai Road, Qingdao 266237, China; 3 College of Earth and Planetary Sciences, University of Chinese Academy of Sciences, Beijing 100049, China

**Keywords:** jellyfish, *Cyanea nozakii*, antivenom, AntiCnTXs, F(ab’)_2_-AntiCnTXs, Fab-AntiCnTXs

## Abstract

Jellyfish stings are a common issue globally, particularly in coastal areas in the summer. Victims can suffer pain, itching, swelling, shock, and even death. Usually, hot water, vinegar, or alumen is used to treat the normal symptoms of a jellyfish sting. However, a specific antivenom may be an effective treatment to deal with severe jellyfish stings. *Cyanea nozakii* often reach a diameter of 60 cm and are responsible for hundreds of thousands of stings per year in coastal Chinese waters. However, there has been no specific *C. nozakii* antivenom until now, and so the development of this antivenom is very important. Herein, we collected *C. nozakii* antisera from tentacle extract venom immunized rabbits and purified the immunoglobulin (IgG) fraction antivenom (AntiCnTXs). Subsequently, two complete procedures to produce a refined F(ab’)_2_ type of antivenom (F(ab’)_2_-AntiCnTXs) and Fab type of antivenom (Fab-AntiCnTXs) by multiple optimizations and purification were established. The neutralization efficacy of these three types of antivenoms was compared and analyzed in vitro and in vivo, and the results showed that all types of antibodies displayed some neutralization effect on the lethality of *C. nozakii* venom toxins, with the neutralization efficacy as follows: F(ab’)_2_-AntiCnTXs ≥ AntiCnTXs > Fab-AntiCnTXs. This study describes the preparation of novel *C. nozakii* jellyfish antivenom preparations towards the goal of developing a new, effective treatment for jellyfish stings.

## 1. Introduction

Venomous animal bites or stings pose a major threat to human beings. Snake bites, spider stings, scorpion stings, jellyfish stings, etc. cause many deaths every year [1]. Jellyfish stings are a common issue globally in coastal areas in the summer. Victims can suffer pain, itching, swelling, shock, and even death [2,3]. Usually, a hot water compress, vinegar, alumen solution, or seawater rinsing are used as first aid to alleviate pain or prevent further discharge of the unfired nematocysts remaining on the skin in the case of a mild jellyfish sting. However, some treatments, such as seawater rinsing, have actually been proven to increase the venom load [4,5,6]. For severe jellyfish stings, a more effective treatment is needed. Zinc gluconate inhibited potassium efflux and prolonged survival time in mice and MβCD, while HPβCD suppressed tissue necrosis and pain in mice after box jellyfish envenomation [7,8]. Moreover, a specific antivenom may also be an additional therapeutic approach to deal with severe jellyfish stings. *Cyanea nozakii* jellyfish often reach a diameter of 60 cm and are responsible for hundreds of thousands of stings per year in coastal Chinese waters. Unfortunately, many deaths from jellyfish stings have been reported in China in recent years. However, there has been no effective method to treat severe *Cyanea nozakii* stings; dexamethasone, aspirin, and antihistamines cannot stop victims’ systemic symptoms in clinic [9]. Therefore, the development of a *C. nozakii* antivenom is urgent.

In general, antivenom has effectively neutralized venom toxins and saved thousands of lives since the 19th century [10,11]. Historically, whole antiserum was used to neutralize the toxins. However, this contains not only antitoxins but also many other proteins, which may cause some potential side effects after injection into the body. In 1937, the γ globulin (immunoglobulin G, IgG) was discovered to be the antitoxin in the antiserum [12,13,14,15]. The purified IgG without other serum proteins was then used as a second-generation antitoxin. IgG is composed of two light chains and two heavy chains connected by disulfide bonds and contains a fragment of antigen-binding domain (Fab) and fragment crystallized domain (Fc) [16]. As most antiserums are produced by animals such as horses and rabbits, the heterogenous Fc domain may cause an immunological reaction in the body, and many serum sicknesses have also been reported after injection of antivenom, including a previously sheep-sourced and IgG type of box jellyfish antivenom [17,18,19]. Therefore, the removal of the Fc fragment from IgG not only preserves the function of antigen-binding but also decreases the potential serum sickness of the heterogenous Fc domain. 

Two types of Fc fragment that remove IgG, F(ab’)_2_, and Fab are available. Both F(ab’)_2_ and Fab types of antivenom have been successfully used in snakebite treatment. F(ab’)_2_ antivenom has two Fab domains; is very similar to the whole IgG in structure; and can form multivalent immunocomplexes with toxin antigens, such as IgG, and then be cleaned by phagocytic cells. Fab antivenom only has one Fab domain with a smaller molecular weight and can be easily distributed to the whole body. However, Fab antivenom cannot work as F(ab’)_2_ or IgG antivenom. Currently, commercial snake, scorpion, spider, stonefish IgG, F(ab’)_2_, and Fab antivenom are available for emergency treatment (Table 1). 

However, all of these commercial antivenoms can lead to side effects, such as headache, fever, nausea, swollen glands, chest tightness, pounding heartbeats, or trouble breathing. So, it is hard to say which kind of antivenom is better, especially for the envenomation of different animals. To date, only one jellyfish antivenom, the Commonwealth Serum Laboratories™ (CSL) box jellyfish antivenom, has been available for the treatment of box jellyfish stings worldwide, but this antivenom does not reliable to prevent death; it in fact lessens the survival time in mice [7,20]. Additionally, the venom components are very different between box jellyfish *Chironex fleckeri* and *Cyanea nozakii* [21,22,23,24], which means that CSL box jellyfish antivenom is not suitable to deal with a *C. nozakii* jellyfish sting. Furthermore, no evidence shows that *C. fleckeri* antivenom has any efficacy in nonchirodropid box jellyfish stings [25,26].

In the present study, we prepared a *C. nozakii* antivenom in rabbits by immunizing rabbits with an extract preparation of tentacle venom comprising venom toxins and other tentacle components. IgG (AntiCnTXs) was isolated by Protein A resin from the antiserum. Subsequently, AntiCnTXs were refined to F(ab’)_2_ type of antivenom (F(ab’)_2_-AntiCnTXs) by pepsin and Fab type of antivenom (Fab-AntiCnTXs) by papain, respectively. We then compared their neutralization efficacy on CnTXs both in vitro and in vivo. All kinds of antivenom showed some neutralization effect on the lethality of CnTXs in an in vivo experiment, and the neutralization efficacy was as follows: F(ab’)_2_-AntiCnTXs ≥ AntiCnTXs > Fab-AntiCnTXs. Furthermore, our study provides important information for the preparation of different antivenoms for the treatment of *C. nozakii* jellyfish stings in the future.

## 2. Results

### 2.1. Affinity Purification of AntiCnTXs from Antiserum

**Figure 1:**
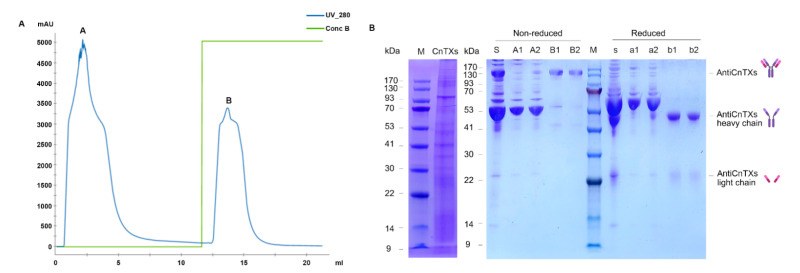
Purification of IgG-AntiCnTXs from the antiserum. (**A**) Protein A affinity purification of AntiCnTXs from antiserum. (**B**) SDS-PAGE analysis of CnTXs and the fractions from protein A affinity purification. M, protein markers; CnTXs, the antigen used for preparation of antivenom; S, antiserum under the nonreduced condition; A1 and A2, fractions of peak A under the nonreduced condition; B1 and B2, fractions of peak B under the nonreduced condition; s, antiserum under the reduced condition; a1 and a2, fractions of peak A under the reduced condition; b1 and b2, fractions of peak B under the reduced condition.

The crude serum contains many blood proteins and immunoglobulins. A Protein A column can separate IgG of AntiCnTXs from other protein with very high affinity (Figure 1). Most other proteins do not bind the resin and wash out in the flow-through fraction. However, AntiCnTXs can bind to the resin and are eluted with elution buffer B. The SDS-PAGE profile (Figure 1B) indicates that the purity of AntiCnTXs is very high, and there is only one protein band with a molecular weight of ~150 kDa under the nonreduced condition. IgG is composed of two heavy chains and two light chains; however, the disulfide bonds in the IgG are broken under the reduced condition and then generate two separate heavy chains and two separate light chains.

### 2.2. Preparation of F(ab’)2 Fragment of AntiCnTXs

#### 2.2.1. Optimization of Pepsin Digestion of AntiCnTXs

**Figure 2:**
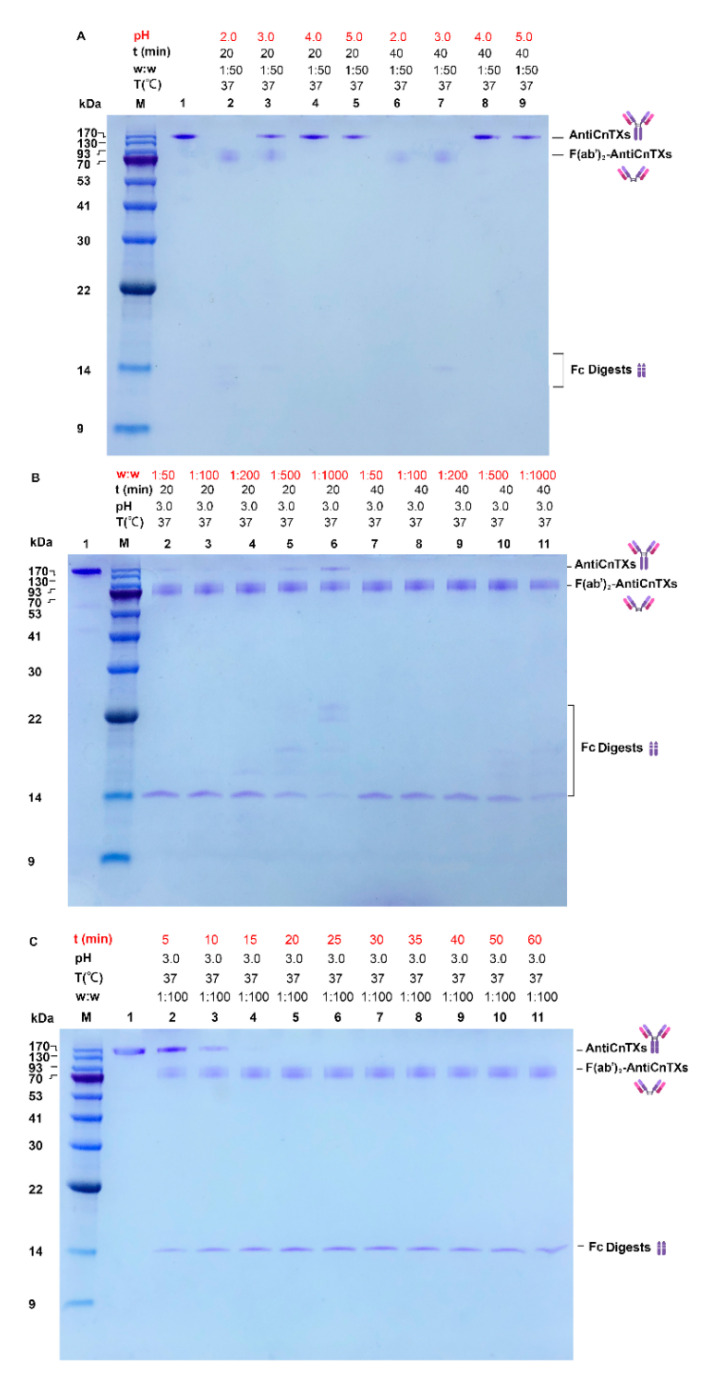
SDS-PAGE analysis of the optimal conditions screen for the pepsin digestion of AntiCnTXs. (**A**) The effect of pH on the pepsin digestion of AntiCnTXs; (**B**) the effect of w:w (pepsin:AntiCnTXs) on the pepsin digestion of AntiCnTXs; (**C**) the effect of reaction time on the pepsin digestion of AntiCnTXs. The details of digestion conditions are listed for each lane.

The reaction system is very important to the pepsin digestion of AntiCnTXs. pH is among the most critical factors for the enzyme. As seen in Figure 2, the SDS-PAGE profile shows the screening of optimal reaction conditions for the pepsin digestion of AntiCnTXs. In Figure 2A, all the AntiCnTXs can be digested by pepsin at pH 2.0 in 20 min and about half at pH 3.0, and pepsin can digest all the AntiCnTXs at pH 3.0 in 40 min. However, almost none is digested at pH 4.0 or 5.0, even after 40 min. So, pepsin activity is highest at pH 2.0. Given the extreme condition at pH 2.0 for AntiCnTXs, pH 3.0 is not only much milder but also very effective for AntiCnTXs digestion. Therefore, pH 3.0 is more suitable for the pepsin digestion of AntiCnTXs. Figure 2B shows the pepsin to AntiCnTXs ratio for digestion, and the SDS-PAGE profile displays that W_pepin_:W_AntiCnTXs_ = 1:50 to 1:200 is very effective for pepsin to cleave AntiCnTXs into F(ab’)_2_-AntiCnTXs and Fc digests. The reaction time assay shows that AntiCnTXs can be almost fully digested by pepsin in 15 min at 37 °C at a ratio of 1:100 (Figure 2C). So, to ensure that the AntiCnTXs are totally digested by pepsin, the reaction conditions for pepsin digestion of AntiCnTXs are pH 3.0, W_pepin_:W_AntiCnTXs_ = 1:100, and 30 min for the following preparation of F(ab’)_2_-AntiCnTXs.

#### 2.2.2. Purification of F(ab’)_2_-AntiCnTXs

**Figure 3:**
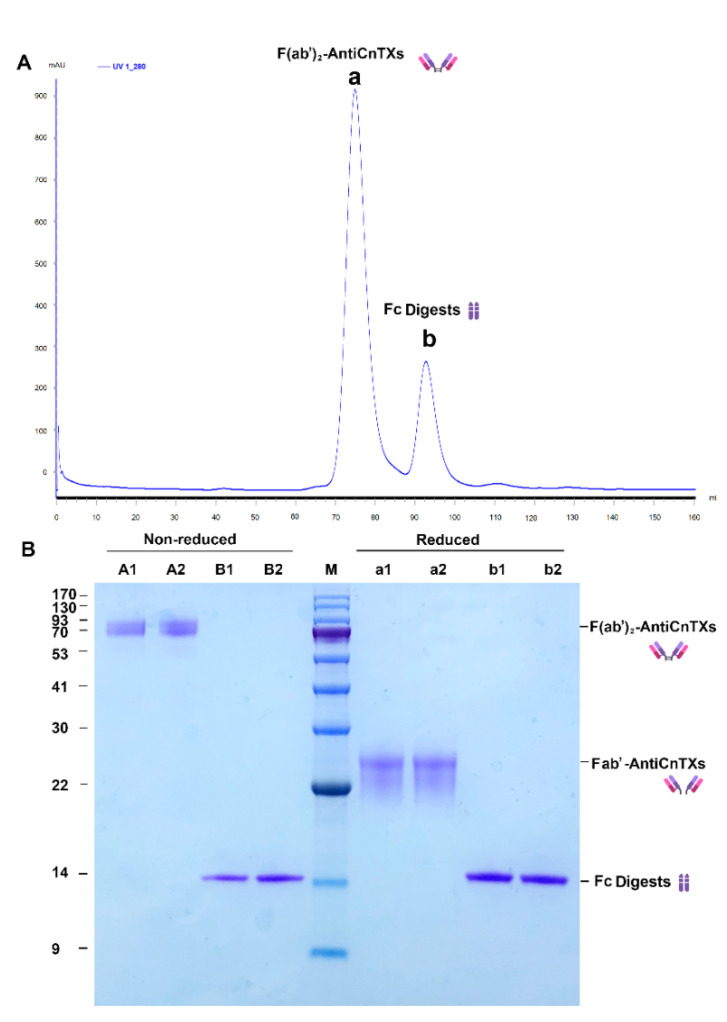
Purification of F(ab’)_2_-AntiCnTXs from the pepsin digests of AntiCnTXs. (**A**) Size exclusion chromatography purification of F(ab’)_2_-AntiCnTXs from the pepsin digests of AntiCnTXs. (**B**) SDS-PAGE analysis of the fractions from size exclusion chromatography purification. A1 and A2, peak a under the nonreduced condition; B1 and B2, peak b under the nonreduced condition; M, protein markers; a1 and a2, peak a under the reduced condition; b1 and b2, peak b under the reduced condition.

AntiCnTXs digests were purified by size exclusion chromatography HiLoad Superdex 200 16/60. Figure 3A shows that only two protein peaks were achieved in the chromatogram, and the SDS-PAGE profile of the two peaks displays very good purity in Figure 3B. The molecular weight of F(ab’)_2_-AntiCnTXs was about 90 kDa the under nonreduced condition; however, a ~26 kDa band was observed under the reduced condition because the disulfide bond was broken.

### 2.3. Preparation of Fab Fragment of AntiCnTXs

#### 2.3.1. Optimization of Papain Digestion of AntiCnTXs

**Figure 4:**
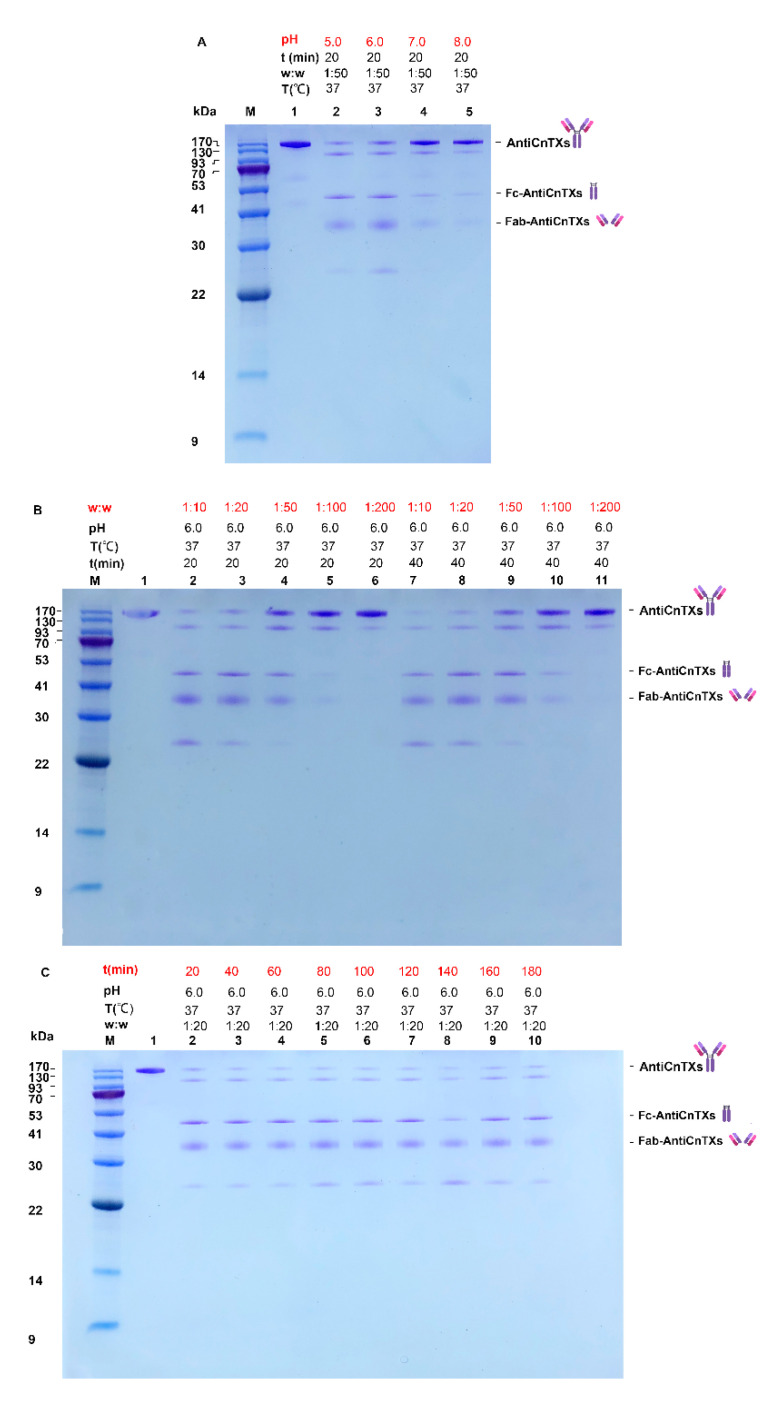
SDS-PAGE analysis of the optimal conditions screen for the papain digestion of AntiCnTXs. (**A**) The effect of pH on the papain digestion of AntiCnTXs; (**B**) the effect of w:w (papain: AntiCnTXs) on the papain digestion of AntiCnTXs; (**C**) the effect of reaction time on the papain digestion of AntiCnTXs. The details of digestion conditions are listed in each lane.

Reaction condition is very important to the papain digestion of AntiCnTXs. pH 5.0 or 6.0 is much more effective than pH 7.0 or 8.0 (Figure 4A) for papain digestion. The SDS-PAGE profile displays that W_Papain_:W_AntiCnTXs_ = 1:10‒1:20 is more effective for pepsin to cleave AntiCnTXs into Fab-AntiCnTXs and Fc digests (Figure 4B). Figure 4C shows that papain is not very effective at digesting AntiCnTXs; they are not totally digested even after 180 min. The reaction conditions for papain digestion of AntiCnTXs are pH 6.0, W_papain_:W_AntiCnTXs_ = 1:200, and 60 min for the following preparation of Fab-AntiCnTXs.

#### 2.3.2. Purification of Fab-AntiCnTXs

**Figure 5:**
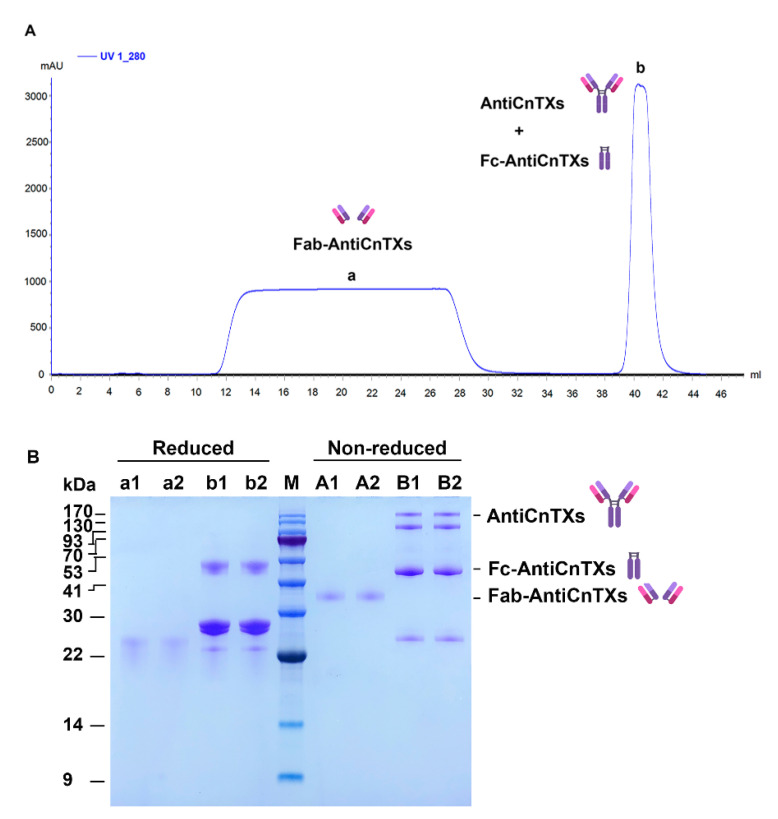
Purification of Fab-AntiCnTXs from the papain digests of AntiCnTXs. (**A**) Protein A affinity purification of Fab-AntiCnTXs from the papain digests of AntiCnTXs. (**B**) SDS-PAGE analysis of the fractions from Protein A affinity purification of Fab-AntiCnTXs. A1 and A2, peak a under the nonreduced condition; B1 and B2, peak b under the nonreduced condition; M, protein markers; a1 and a2, peak a under the reduced condition; b1 and b2, peak b under the reduced condition.

As the papain digests of AntiCnTXs contain Fab-AntiCnTXs, Fc-AntiCnTXs, and some undigested AntiCnTXs, a Protein A column was used to separate Fab-AntiCnTXs from other proteins. Figure 5A shows the chromatogram of the purification. The SDS-PAGE profile indicates that the purity of Fab-AntiCnTXs is very good in Figure 5B. The molecular weight of Fab-AntiCnTXs was about 36 kDa under nonreduced conditions; however, the disulfide bond between two Fab-AntiCnTXs was broken by βME under reduced conditions.

### 2.4. Neutralization Assay of the Antivenoms

**Figure 6:**
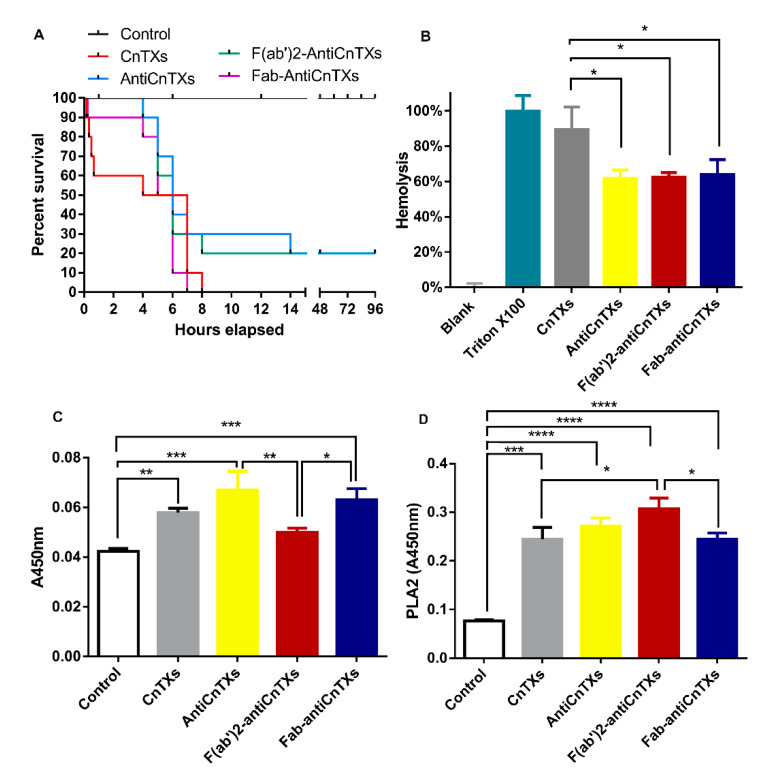
Neutralization assay of the antivenoms against the toxicities of CnTXs. (**A**) Neutralization assay of AntiCnTXs, F(ab’)_2_-AntiCnTXs, and Fab-AntiCnTXs against the lethality of CnTXs in vivo. Control, injection of dialysis buffer; CnTXs, injection of CnTXs; F(ab’)_2_-AntiCnTXs: injection of F(ab’)_2_-AntiCnTXs neutralized CnTXs; Fab-AntiCnTXs, injection of Fab-AntiCnTXs neutralized CnTXs, *n* = 10. (**B**) Neutralization assay of antivenoms against the hemolytic activity of CnTXs in vitro; (**C**) neutralization assay of antivenoms against the metalloprotease activity of CnTXs in vitro; (**D**) neutralization assay of antivenoms against the PLA_2_ activity of CnTXs in vitro; * *p* < 0.05, ** *p* < 0.03, *** *p* < 0.0003, **** *p* < 0.0001, *n* = 3.

The efficacy of these antibodies and IgG fragments to neutralize venom toxins was evaluated using in vivo and in vitro assays. The in vivo assay results show that all the mice died within 8 h after intraperitoneal injection of CnTXs and 40% died within 40 min. However, the mice in the antibody-neutralized groups died much later than those in the CnTXs group (Figure 6A). Moreover, 20% of mice survived in both the AntiCnTXs and F(ab’)_2_-AntiCnTXs groups. However, all the mice in the Fab-AntiCnTXs group died within 8 h after injection (Figure 6A), which indicated that the neutralization of Fab-AntiCnTXs was less effective than that of AntiCnTXs or F(ab’)_2_-AntiCnTXs. PLA_2_, hemolytic, and metalloprotease activity are among the most obvious toxicities of CnTXs in vitro. All kinds of antibodies, AntiCnTXs, F(ab’)_2_-AntiCnTXs, and Fab-AntiCnTXs, significantly inhibited the hemolytic activity of CnTXs (Figure 6B). However, no inhibitory effect was observed in the PLA_2_ and metalloprotease activity assay. In contrast, it could promote PLA_2_ and metalloprotease activity in vitro (Figure 6C,D).

### 2.5. LC-MS/MS and GO Analysis of Antivenom

**Figure 7:**
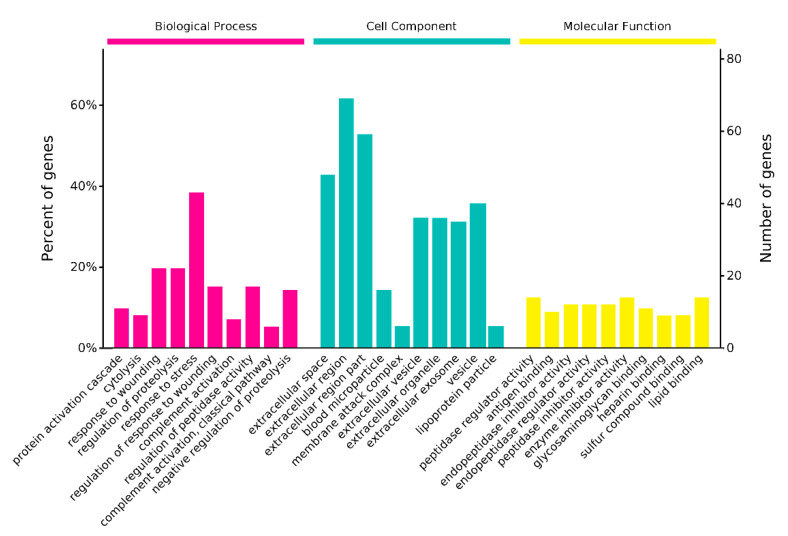
LC-MS/MS and GO analysis of antiserum. All the identified proteins were summarized in three categories: cellular component, biological process, and molecular function.

The LC-MS/MS analysis of CnTXs antiserum identified 130 proteins in total (Table S1). The CnTXs antiserum contains many immune molecules, including IgG, a membrane attack complex, to resist the invasion of CnTXs (Figure 7). All the IgGs are very similar in the structure, such as the Y shape and Fc domain. However, the Fab domains of those IgGs are quite different from each other, so they can bind to different antigens and act as protein inhibitors. So, in the molecular function analysis, many protein inhibitors and antigen-binding proteins were identified, and that is why the antibody can neutralize the antigen CnTXs.

## 3. Discussion

Antivenom is a good way to treat venomous animal bites or stings, and so is highly recommended as first aid by the World Health Organization. Terrestrial venomous animals, such as snakes, scorpions, spiders, and bees, pose a threat to human beings. Antivenoms have already been well studied and developed over many years to treat bites and stings [27,28,29]. Snake antivenom is the most successful example, as it has been widely used for centuries and has saved hundreds of thousands of lives all over the world. There are also many venomous marine animals, including sea snakes, jellyfish, stonefish, blue-ringed octopus, cone snails, pufferfish, and ciguatoxin-containing fishes. However, only certain sea snakes, the box jellyfish *Chironex fleckeri*, and stonefish have antivenom to date [26,30], and many people have died from a lack of effective medicine, such as antivenom; deaths have also occurred, despite the administration of antivenoms sometimes. So, the development of marine antivenom is very important.

Antivenom is composed of many antibodies for the neutralization of animal toxins. Nowadays, both traditional IgG antivenom and F(ab’)_2_ or Fab type of refined antivenoms are produced by antivenom manufacturers (Table 1). However, it is difficult to balance the efficacy of IgG and its side effects. The best way is to analyze the neutralization effectiveness of all three types of antivenom for different animal antivenoms. Therefore, in the current study, we prepared a CnTXs jellyfish antivenom in rabbits and then purified CnTXs antibodies from the antiserum and made refined antibodies. Usually, pepsin and papain are used to produce F(ab’)_2_ and Fab types of antivenom, respectively [31,32,33,34,35,36]. So, we optimized multiple reaction conditions for the digestion of CnTXs by pepsin or papain and finally produced two types of refined CnTXs antibodies, F(ab’)_2_-AntiCnTXs and Fab-AntiCnTXs. However, F(ab’)_2_-AntiCnTXs digestion by pepsin was much faster and simpler than Fab-AntiCnTXs prepared by papain. Pepsin could also digest almost 100% of AntiCnTXs in 30 min with a ratio of 1:100. However, papain only digested about 90% of AntiCnTXs in 3 h with 5-fold more enzyme than pepsin. So, pepsin is much more effective than papain at removing the Fc domain from IgG AntiCnTXs. Furthermore, the in vivo neutralization efficacy of Fab-AntiCnTXs is much worse than that of AntiCnTXs or F(ab’) _2_-AntiCnTXs. This is because Fab-AntiCnTXs could not form multivalent immunocomplexes with toxins, but AntiCnTXs and F(ab’)_2_-AntiCnTXs could. This may be why most commercial antivenoms are of the IgG or F(ab’)_2_ type instead of the Fab type. 

Our previous study showed that CnTXs are composed of many types of toxins, including phospholipase A_2_, metalloproteinase, serine protease inhibitor, plancitoxin-1, and alpha-latrocrustotoxin-Lt1a [21], and metalloproteinase might be the key lethal toxin in the venom of *Cyanea nozakii* [37]. In the present study, the LC-MS/MS analysis of the CnTXs antiserum identified many proteinase inhibitors, which indicated that the Fab domain of some AntiCnTXs is homologous with proteinase inhibitors in structure and may inhibit the proteinases in the venom. So, in the neutralization assay, the mice that were treated with F(ab’) _2_-AntiCnTXs or AntiCnTXs preincubated with the tentacle venom extract died much later than the mice injected with the tentacle venom extract alone, and 20% of mice survived in both groups. No commercial antivenom is a guaranteed lifesaver. However, the survival rate of F(ab’) _2_-AntiCnTXs and AntiCnTXs treatment is still not very high. The low proportion of lethal toxins in CnTXs may be among the reasons for this. CnTXs, used for the preparation of antivenom, are complex mixtures that contain many other nontoxic proteins and do not represent the real toxins injected in an authentic sting. It is hard to extract pure jellyfish venom toxins, as with snakes and spiders. Jellyfish toxins are in the tentacle nematocyst. The sonication, glass bead disruption, or high-pressure cell rupture of isolated nematocysts is often used for the extraction of jellyfish toxins, and the whole extract is then used as jellyfish toxin for the research [7,38,39,40]. Therefore, these “jellyfish toxins” are composed of toxins and many other nontoxic nematocyst proteins. The nontoxins’ antibodies in the antivenom may affect the neutralization efficacy. Moreover, the antivenom is a mixture of rabbit immunoglobulins, which are heterologous proteins to humans and may also be recognized by the human immune system as antigens. Although real jellyfish toxins’ antibodies could neutralize these toxicities, other nontoxins’ antibodies in the antivenom may become toxic to the body. The antivenoms we produced in this study may not be suitable for the treatment of real jellyfish stings, and further studies will be needed to improve the efficacy and safety of antivenom, including collecting and using pure jellyfish venom [7,41] or purified lethal toxins as antigen to prepare antivenom to neutralize the jellyfish toxins and minimize potential side effects.

## 4. Conclusions

In the current study, a *C. nozakii* jellyfish antivenom was prepared in rabbits and refined AntiCnTXs into F(ab’)_2_-AntiCnTXs by pepsin and Fab-AntiCnTXs by papain, respectively. The neutralization efficacy of these three types of antivenom was compared and analyzed both in vitro and in vivo. The results showed that the neutralization effect on the lethality of CnTXs was as follows: F(ab’)_2_-AntiCnTXs ≥ AntiCnTXs > Fab-AntiCnTXs. Future research on more effective *C. nozakii* jellyfish antivenom still needs to be performed, using purified toxins as antigens. Moreover, an animal model will also be set up using live tentacles to model an authentic sting to assess the efficacy of *C. nozakii* antivenom. This study not only provides useful information on the preparation of a *C. nozakii* jellyfish antivenom but also offers new insights to produce other marine antivenoms in the future.

## 5. Materials and Methods

### 5.1. Jellyfish Specimen Collection and Toxin Preparation

*Cyanea nozakii* specimens were collected from the coast of Qingdao, China, in 2019. The fresh tentacles were cut from the body and stored at −80 °C. The frozen tentacles were autolyzed at 4 °C for 12–24 h, and the undissolved samples were removed with a plankton net. Finally, the autolyzed solution was centrifuged at 10,000× *g* for 15 min at 4 °C, and the supernatant containing jellyfish toxins was used as *C. nozakii* toxins (CnTXs). 

### 5.2. Animal Immunization and Antiserum Preparation

Firstly, the lethality of CnTXs was tested to make sure that it contained jellyfish toxins to produce their antibodies. Subsequently, the toxicity of CnTXs was attenuated so that the rabbits were not killed in the immunization process. The attenuation of CnTXs was as follows: 40% formaldehyde was added to CnTXs at a ratio of 1:50 and then incubated at 37 °C for a week; 40% formaldehyde was added again into the mixture at a ratio of 1:200 with incubation at 37 °C for another week. Subsequently, it was dialyzed against 20 mM PBS, pH 7.0, to remove the formaldehyde. Finally, after centrifugation, the supernatant was filtered with a 0.22 µm filter and kept in a −80 °C freezer.

Attenuated CnTXs (0.68 mg, 0.43 mg/mL), together with complete Freund’s adjuvant, was injected into three healthy New Zealand white rabbits (~2 kg) as the first immunization. The second immunization was performed three weeks later using 0.34 mg attenuated CnTXs and incomplete Freund’s adjuvant, and the third and fourth immunizations were performed two weeks after the previous immunization, using 0.34 mg attenuated CnTXs and incomplete Freund’s adjuvant. The final immunization was completed three weeks after the fourth immunization. A titer test of the antiserum was performed after the fourth and fifth immunizations. Briefly, the titer of the antiserum was evaluated using the ELISA method. The antigen of attenuated CnTXs was coated in a microtiter palate with a coating buffer (50 mm, pH 9.6, Na_2_CO_3_) at 4 °C overnight. After removing the coating buffer, the plate was washed with 0.05% Tween-20, 20 mM NaH_2_PO_4_, pH 7.4 (PBST) three times, followed by blocking with 5% skim milk for 1 h. After another three washes with PBST, various diluted antiserums were added and incubated at 37 °C for 1 h, with three washes after. Then, HRP-labeled Goat Antirabbit IgG (H+L) was used and incubated at 37 °C for 45 min. The plate was again washed three times with PBST. Subsequently, 3’3’5’5’-tetramethylbenidine dihydrochloride (TMB) substrate was added, and H_2_SO_4_ was used to stop the reaction 15 min later. Finally, the absorbance was recorded at 450 nm.

Once the titer test of the antiserum was qualified, the whole blood was collected and the antiserum was prepared by centrifugation at 4 °C and 3000 rpm for 15 min. Finally, the antiserum was stored at −80 °C until further use. All animals in this experiment received humane care, as approved by the Ethics Committee of the Institute of Oceanology, Chinese Academy of Sciences; approval code: IOCAS/KLEMB/20180309; approval date: 9 March 2018.

### 5.3. Purification of CnTXs Antibody

The antiserum was diluted with binding buffer A (0.15M NaCl, pH 7.0, 20 mM Na_2_HPO_4_) at a ratio of 1:1 (v:v) and purified with a fast protein liquid chromatogram system ÄKTA pure (GE Healthcare, Chicago, IL, USA), equipped with a 1 mL Protein A prepacked column (GenScript, Piscataway, NJ, USA), and monitored at 280 nm. Buffer A was used to wash and remove the unbound antiserum proteins, and Buffer B (pH = 3.0, 100 mM glycine) was used to elute the antibodies of CnTXs (AntiCnTXs). The elution was immediately adjusted with 1 M Tris-HCl, pH8.5. The purity of flow-through and elution were analyzed by SDS-PAGE under both nonreduced and reduced conditions.

### 5.4. Refinement of AntiCnTXs

#### 5.4.1. F(ab’)_2_ Fragments of AntiCnTXs Preparation

##### The Optimum Screen of Pepsin Digestion of AntiCnTXs

The optimum pH for the pepsin digestion was conducted as follows: aliquots of AntiCnTXs were dialyzed at 4 °C overnight against dialysis buffers of pH 2.0, pH 3.0, pH 4.0, pH 5.0, and 100 mM glycine, respectively. Equal pepsin was added into 100 μL AntiCnTXs at different pH values at the same ratio of W_pepsin_:W_AntiCnTXs_ = 1:50, followed by incubation at 37 °C for 20 and 40 min, with three replicates. Subsequently, the digested AntiCnTXs were analyzed by SDS-PAGE under the nonreduced conditions. The optimum ratio of Wpepsin: WAntiCnTXs for the digestion was determined as follows: pepsin was added to the same AntiCnTXs to a final ratio of 1:50, 1:100, 1:200, 1:500, or 1:1000. The reaction was carried out at pH 3.0, 37 °C for 20 min with three replicates. Finally, the digested AntiCnTXs were analyzed by SDS-PAGE under the nonreduced conditions. The optimum time for digestion was determined as follows: AntiCnTXs were digested by pepsin at a ratio of 1:100 at pH 3.0 and 37 °C for 5, 10, 15, 20, 25, 30, 35, 40, 50, or 60 min, with three replicates. The digests were immediately quenched at 95 °C for 5 min once time was up. Finally, the digested AntiCnTXs were analyzed by SDS-PAGE under the nonreduced conditions.

##### Purification of F(ab’)_2_-AntiCnTXs

AntiCnTXs digestion by pepsin was scaled up at pH 3.0, W_pepsin_:W_AntiCnTXs_ = 1:100 for 30 min. The digests were concentrated with concentrators (MWCO 10 kDa, Millipore, Burlington, MA, USA) at 6000× *g*, 4 °C, and then loaded onto a HiLoad Superdex 200 16/60 column (GE Healthcare) with buffer A. The purity was analyzed by SDS-PAGE under both nonreduced and reduced conditions.

#### 5.4.2. Fab Fragments of AntiCnTXs (Fab-AntiCnTXs) Preparation

##### The Optimum Screen of Papain Digestion of AntiCnTXs

The optimum pH for the papain digestion was determined as follows: aliquots of AntiCnTXs were dialyzed at 4 °C overnight against dialysis buffers of pH 5.0, 100 mM glycine, pH 6.0, pH 7.0, pH 8.0, and 20 mM PBS. The same amount of papain was added into 100 μL AntiCnTXs at different pH values at the same ratio of W_papain_:W_AntiCnTXs_ = 1:50, followed by incubation at 37 °C for 20 min, with three replicates. Subsequently, the digested AntiCnTXs were analyzed by SDS-PAGE under the nonreduced conditions. The optimum ratio of W_papain_:W_AntiCnTXs_ for the digestion was determined as follows: papain was added to the same amount of AntiCnTXs to a final ratio of 1:10, 1:20, 1:50, 1:100, or 1:200. The reaction was carried out at pH 3.0, 37 °C for 20 and 40 min, with three replicates. Finally, the digested AntiCnTXs were analyzed by SDS-PAGE under the nonreduced conditions. The optimum time for the digestion was determined as follows: AntiCnTXs were digested by papain at a ratio of 1:20 at pH 6.0, 37 °C for 20, 40, 60, 80, 100, 120, 140, 160, or 180 min, with three replicates. The digests were immediately quenched at 95 °C for 5 min once time was up. Finally, the digested AntiCnTXs were analyzed by SDS-PAGE in nonreduced conditions.

##### Purification of Fab-AntiCnTXs

AntiCnTXs digestion by pepsin was scaled up under the optimized conditions of pH 6.0, W_papain_:W_AntiCnTXs_ = 1:20 for 60 min. The digests were again purified by a Protein A column to remove the Fc fragments and undigested AntiCnTXs. The purity of Fab-AntiCnTXs was analyzed by SDS-PAGE under both nonreduced and reduced conditions.

### 5.5. Neutralization Assay of the Antivenoms

#### 5.5.1. In Vivo Neutralization Assay of the Antivenom

SPF KM mice (18–20 g) were used for in vivo neutralization assay. Each group contained 10 mice, five males and five females. AntiCnTXs, F(ab’)_2_-AntiCnTXs, Fab-AntiCnTXs, and CnTXs were dialyzed in a dialysis buffer (20 mM, Tris-HCl, pH 7.0, 0.15 M NaCl) at 4 °C overnight. The concentration of each sample was measured using the Bradford method [42]. A total of 700 μL mixture containing 330 μg CnTXs and 330 μg AntiCnTXs, 198 μg F(ab’)_2_-AntiCnTXs, or 86 μg Fab-AntiCnTXs was incubated to neutralize the toxicity of CnTXs at 4 °C for 1 h. Then, 700 μL neutralized CnTXs and the same amount of unnaturalized CnTXs were intraperitoneally injected into each mouse using a dialysis buffer as a control. The mortality was recorded over the next 98 h. All animal experiments in this study were approved by the Ethics Committee of the Institute of Oceanology, Chinese Academy of Sciences.

#### 5.5.2. In Vitro Hemolysis Activity Neutralization Assay

In vitro neutralization efficacy on the hemolysis activity of CnTXs was assayed using a previous method with some modifications [37]. In brief, 25 μg CnTXs, 25 μg AntiCnTXs, 15 μg F(ab’)_2_-AntiCnTXs, or 6.5 μg Fab-AntiCnTXs was incubated to neutralize the toxicity of CnTXs and then diluted to 100 μL with 0.9% NaCl. Then, 200 μL human erythrocyte suspended was then added and incubated at 37 °C for 30 min using an isotonic buffer and Triton X-100 as the blank and positive control, respectively. After centrifugation at 3000 rpm for 10 min, the hemoglobin released in the supernatant was assayed at 405 nm. The hemolysis rate was calculated as (A_405_(sample) − A_405_(blank))/(A_405_(Triton X-100) − A_405_(blank)) × 100%. All the experiments were conducted with three replicates.

#### 5.5.3. In Vitro Phospholipase A_2_ (PLA_2_) Activity Neutralization Assay

The neutralization efficacy of this antivenom on PLA_2_ activity was measured according to a method described before [37]. Briefly, 25 μg CnTXs, 25 μg AntiCnTXs, 15 μg F(ab’)_2_-AntiCnTXs, or 6.5 μg Fab-AntiCnTXs was incubated to neutralize the toxicity of CnTXs, respectively, and then diluted to 250 μL with 50 mM Tris-HCl, 5 mM CaCl_2_, 100 mM NaCl, pH 8.0, followed by the addition of 25 μL, 1 mg/mL 4-nitro-3-octanoyloxybenzoic acid (NOBA); 50 mM Tris-HCl, 5 mM CaCl2, 100 mM NaCl, pH 8.0, and CnTXs were used as controls. Subsequently, the plate was incubated at 37 °C for 1 h, and the absorbance was measured at 405 nm. All the experiments were conducted with three replicates.

#### 5.5.4. In Vitro Metalloproteinase Activity Neutralization Assay

The neutralization efficacy of this antivenom on metalloproteinase activity was assayed according to a previous method [37]. Briefly, 25 μg CnTXs, 25 μg AntiCnTXs, 15 μg F(ab’)_2_-AntiCnTXs, or 6.5 μg Fab-AntiCnTXs was incubated to neutralize the toxicity of CnTXs; then diluted to 100 μL with 50 mM Tris-HCl, pH 8.8, 5 mM CaCl_2_, 150 mM NaCl; followed by the addition of 100 μL of 5 mg/mL Azocasein and incubation at 37 °C for 90 min. The reactions were quenched by the addition of 200 μL of 0.5M trichloroacetic acid and placed at room temperature for 30 min. The precipitate was removed by centrifugation at 10,000 rpm for 10 min. Finally, 150 μL supernatant was neutralized with 150 μL 0.5 M NaOH, and the absorbance was measured at 450 nm. All the experiments were conducted with three replicates.

### 5.6. LC-MS/MS and GO Analysis of Antivenom

LC-MS/MS analysis of the antiserum was conducted according to a previous method [21]. Briefly, all the antiserum proteins in the SDS-PAGE gel were cut off and then destained by 25 mM NH_4_HCO_3_, 50% acetonitrile (ACN), followed by dehydration by 50% and 100% ACN for 30 min, separately. Next, 10 mM DTT or 25 mM NH_4_HCO_3_ was used at 57 °C for 1 h. Subsequently, the sample was treated at room temperature with 50 mM iodoacetamide and 25 mM NH_4_HCO_3_ for 30 min, 25 mmol/L NH_4_HCO_3_ for 10 min, 10 mM DTT and 25 mM NH_4_HCO_3_ for 30 min, and 50 mM iodoacetamide and 25 mM NH_4_HCO_3_ for 30 min in turn. The sample was rehydrated with 10 μL of 0.02 μg/μL trypsin, 25 mM NH_4_HCO_3_, and 10% ACN for 30 min and then 20 μL cover solution for 16 h at 37 °C. The sample was extracted with 50 μL, 5% TFA, and 67% ACN. Finally, the extracted peptides and the supernatant of the gel were combined to dry.

The digested peptides were dissolved in 0.1% formic acid and 2% ACN and analyzed by a C_18_ nanoLC trap column (100 μm × 3 cm, 3 μm, 150 Å) that was washed with 0.1% FA and 2% ACN at 2 μL/min for 10 min, followed by a ChromXP (SCIEX, Framingham, MA, USA) C18 column (75 μm × 15 cm, 3 μm 120 Å) using a gradient of 5–35% CAN, 0.1% FA for 90 min. All the data were acquired from a Triple TOF 5600 system (SCIEX, Framingham, MA, USA) with a Nanospray III source and a pulled quartz tip as the emitter. Instrument parameters were set as ion spray voltage of 2.5 kV, curtain gas of 30 PSI, nebulizer gas of 5 PSI, and an interface heater temperature of 150 °C; 250 ms survey scans were employed for information-dependent acquisition (IDA) with a rolling collision energy setting for all precursor ions. All proteins were matched according to both MS and MS/MS spectra, with ≥95% confidence interval scores in the MASCOT V2.3 (Matrix Science, Inc., Boston, MA, USA) search engine in the database *Oryctolagus cuniculus*. All the identified proteins were annotated in the nonredundant protein database GO (Nr, NCBI) based on the biological process, cell component, and molecular function. 

### 5.7. Statistical Analysis

All the results were expressed as mean ± SD. Statistically significant differences between groups were considered only when *p* < 0.05.

## Figures and Tables

**Table 1:** Species and types of some commercial venomous animals’ antivenoms.

Animal	Species Neutralized	Antivenom Name	Manufacturer	Source	Type
**Snakes**	*Bitis arietans* *Echis ocellatus* *Naja nigricollis*	EchiTab-Plus-ICP	Instituto Clodomiro Picado, Universidad de Costa Rica	Horse	IgG
	*Bungarus fasciatus*, *Bungarus multicinctus*, *Agkistrodon acutus*, *Vipera russelli siamensis*, *Trimeresurus stejnegeri*, *Trimeresurus mucrosquamatus*, *Agkistrodon halys*, *Naja naja atra*, *Ophiophagus Hannah*	+3C	Shanghai Serum Bio-technology Co., LTD, China	Horse	F(ab’)_2_
	*Bitis arietans*, *Bitis gabonica*, *Echis leucogaster*, *Echis ocellatus*, *Echis Pyramidum*, *Dendroaspis polylepis*, *Dendroaspis viridis*, *Naja haje*, *Naja melanoleuca*, *Naja nigricollis*, *Naja pallida*	ANTI-VIPMYN	Instituto Bioclon S.A. de C.V, Mexico	Horse	F(ab’)_2_
	*Bitis arietans*, *Bitis gabonica*, *Echis leucogaster*, *Echis ocellatus*, *Dendroaspis polylepis*, *Dendroaspis jamesoni*, *Dendroaspis viridis*, *Naja haje*, *Naja nigricollis*	FAV-Afrique	Sanofi-Pasteur, France	Horse	F(ab’)_2_
	*Crotalinae subfamily*	CroFab	BTG International, Inc. USA	Sheep	Fab
**Scorpions**	*Centruroides sculpturatus*	Anascorp	Accredo Health Group, Inc. USA	Horse	F(ab’)_2_
	*Androctonus crassicauda* *Androctonus aeneas* *Androctonus australis* *Scorpiomarus palmatus* *Bathus occitanus*	VINS	VINS Bioproducts Limited, India	Horse	F(ab’)_2_
**Jellyfish**	*Chironex fleckeri*	CSL	Commonwealth Serum Laboratories, Limited, Australia	Sheep	IgG
**Spiders**	Red back spiders *Latrodectus hasselti*			Horse	IgG
	Funnel web spider			Rabbit	IgG
**Stonefish**	*Synanceia trachynis*			Horse	IgG

## Data Availability

The data presented in this study are available on request from the corresponding author. The data are not publicly available due to animal ethical reasons.

## Supplementary Materials

The following are available online at https://www.mdpi.com/2072-6651/13/2/165/s1, Table S1: All the proteins identified in the antiserum by LC-MS/MS.
